# Comparative Analysis of Proteomic of Curcumin Reversing Multidrug Resistance in HCT-8/VCR Cells

**DOI:** 10.1155/2022/3605436

**Published:** 2022-04-25

**Authors:** Lei Li, Libo Yu, Xiansheng Cao, Chao Zhang, Qi Liu, Jun Chen

**Affiliations:** ^1^Department of Gastrointestinal Surgery, Yantai Affiliated Hospital of Binzhou Medical University, Yantai, China; ^2^Department of Radiology, Yantaishan Hospital, Yantai, China

## Abstract

To further explore the mechanisms of curcumin reversing multidrug resistance (MDR) in HCT8/VCR cells. Here, we employed comparative analysis of proteomic of essential proteins of human colon carcinoma HCT8/VCR cells with or without treatment of curcumin by separating and quantifying the essential protein posttranslational modification through radical-free two-dimensional polyacrylamide gel electrophoresis with strong reductant. The reverse impact of curcumin on multidrug resistance of HCT8/VCR and HCT8/VCR cells was evaluated using MTT assay. After adding curcumin 25 *μ*M for 72 h, by 2-DE and mass spectrometry, twenty proteins were certified with changed expression levels. Three protein sites were upregulated and seventeen protein sites were downregulated in curcumin-treated HCT-8/VCR. Verification analyses were conducted using RT-PCR and Western blotting for downregulated proteins including GSTP1 and PRDX6. The proteins might have a direct or indirect contact with multidrug resistance. The finding of the research would provide novel sights for systematically comprehending the mechanisms of the reversal impacts of curcumin on MDR in HCT8/VCR cells and contribute to the recognition and application of new markers in clinical practice.

## 1. Introduction

Colorectal carcinoma (CRC) is a high-mortality disease and its morbidity rate prevails in the world, particularly in developed countries. [1]. In Asia, CRC has emerged as the second most common cancer [2]. CRC is the third leading cause of cancer death for both men and women, with an estimated 52 980 persons in the US projected to die of colorectal cancer in 2021 [3]. Although progresses in surgical therapy of CRC, the prognosis of sick persons has not been perfected prominently in the last few decades since most patients are diagnosed with advanced or metastatic cancer. Chemotherapy and radiation therapy in combination with surgical operations would be the most efficient tool to the patients with CRC of intermediate and advanced stage. However, the existence of drug resistance against clinically applied anticarcinoma medicines has been an important barrier in carcinoma treatment [4]. In order to get more efficient chemotherapeutic treatment of CRC sick persons, it is necessary to study the efficient medicines that could reverse MDR. Chemical sensitization such as verapamil, MK571, and CsA could reverse MDR, but they are forbidden in clinical practice because of unacceptable toxicities. As a result, it is indispensable to explore novel reversing drugs that do not have the nonideal toxicological effects [5].

**Figure 1:**
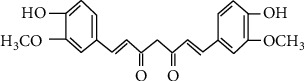
Curcumin chemical structure.

Recently, scientists focus on the traditional Chinese medicine with numerous resource, high effect, low toxicity, and multitarget, and it is able to aim directly to the complex mechanisms of MDR. Recent researches show there will be a bright future on developing the Chinese medicine reversing drugs on cancer MDR. As shown in Figure 1, the polyphenol curcumin (CUR, diferuloylmethane, 1,7-bis-(4-hydroxy-3-methoxyphenyl)-1,6-heptadiene-3,5-dione), an effective constituent for spice turmeric, has been applied as a food additive and a traditional drug in Asia for centuries. There are many biological activities (e.g., antioxidant, anti-inflammatory, antitumor, and antitumorigenesis activities) [6–9]. Curcumin has been reported with the inhibition of P-gp expression in multidrug-resistant human tumor [10, 11], and even reverse the development of every stage of carcinoma [12]. The maximum daily dose of curcumin for an adult is 10 g, while a plasma exposure of 1.77 ± 1.87 mol/L was detected at the dose level of 8 g per day [13]. In our view, curcumin will be a great leading chemical compound for renewing the treatment effect of anticarcinoma medicines without unwished side effects. Currently, comparative proteomics researches of 2-DE are widely applied to chemotherapeutics resistance of many cancer cell lines, including esophageal carcinoma, hepatocellular carcinoma, and breast carcinoma [14–16]. However, global protein analysis is new using 2-DE on reversing of MDR of curcumin in HCT-8/VCR cells.

In this study, we used proteomic skills to study protein extracts from medicine-resistant HCT8/VCR cells and curcumin-treated HCT8/VCR cells. After comparing the expression patterns, proteins with discriminative expressive levels were analyzed using matrix-assisted laser desorption/ionization time-off light mass spectrometry (MALDI-TOF-MS), which was further validated by RT-RCR and Western blotting. To obtain the mechanism of curcumin reversing multidrug resistance of tumor cells and finally find the drug target of multidrug resistance reversing agent, so as to achieve the purpose of reversing multidrug resistance of colon cancer, we have done some preliminary work in this field, which lays a foundation for further study on the mechanism of curcumin reversing multidrug resistance of human colon cancer.

## 2. Materials and Methods

### 2.1. Reagents and Chemicals

Vincristine (VCR) was gained from Main Luck Pharmaceuticals (Shenzhen, China), and curcumin (CUR) and MTT reagent (3-[4,5-dimethylthiazolyl]-2,5-diphenyltetrazolium bromide) were bought from Sigma Chemical Co. (St. Louis, MO, USA). The other reagents applied in 2-DE were provided by Bio-Rad Laboratories (Hercules, CA, USA).

### 2.2. Cell Lines/Cultures

The HCT-8/VCR cell line was gained from the Institute of Biochemistry and Cell Biology (Shanghai, China), which were cultivated in medium containing 10% (vol/vol) fetal bovine serum (Gibco, Rockville, MD, USA) with addition of penicillin (100 U/mL) and streptomycin (100 *μ*g/mL) in Roswell Park Memorial Institute 1640 (Gibco, Rockville, MD, USA). The cell cultures were kept in a moist CO_2_ incubator (5%) under 37°C, and VCR (2 mg/L) was further added to the cell cultures. The cells were cultivated in no medicine culture for 2 weeks before their use in experiments.

### 2.3. MTT Assays

CUR stock solution (1000 mM) was prepared with DMSO, which was diluted by medium to prepare medium of different concentrations of CUR and unified concentration of DMSO (0.1%). HCT-8/VCR cells (5.0 × 10^3^ cells/well) were planted in nighty-six-well plates and incubated for 72 hrs with medium of various CUR concentrations (6.25–100 *μ*M), while controls were incubated with 0.1% DMSO. Afterwards, MTT reagent (20 *μ*L for each well, 5 mg/mL, USB, Austria) was administered and cells were cultivated under 37°C for another 4 hrs. After removal of the medium, DMSO (100 *μ*L each well) was administered, following by gentle plate shaking of 5 minutes. A microplate spectrophotometer (BD Bioscience, Franklin Lakes, NJ, USA) was employed to determine the light absorption value at 570 nm. Every step was performed for three times and each test was performed four times.

### 2.4. Protein Extraction

HCT-8/VCR cells (2 × 10^6^) were treated 25 *μ*M CUR for 72 hours and rinsed with ice-cold PBS buffer solution for three times and directly scraped off from the plates lysed in buffer solution (1 mM PMSF, 1 mM EDTA, 65 mM DTT, 2 M thiourea, 7 M urea, 5 *μ*g/mL RNase, 20 *μ*g/mL DNase, 0.2% (w/v) Bio-Lyte, 4% Chaps). After cultivated on ice for 1 h, centrifugation at 12000 × *g* (4°C, 30 mins) was conducted to separate lysates. The supernatant was transferred and packaged separately and then kept under -80°C to be used. The Bradford protein assay kit (Sangon Biotech, Co, Ltd. Shanghai, China) was employed to determine the protein concentration [17].

### 2.5. Two-Dimensional Gel Electrophoresis

200 *μ*g protein was diluted to 380 *μ*L with rehydration liquor and used to pH 3–10 (nonlinear, 17 cm) IPG strip (Bio-rad, Hercules, CA, USA) by 15-h rehydration (0.001% bromophenol blue, 0.2% IPG buffer, 4% CHAPS, 65 mM DTT, 2 M thiourea, 7 M urea) at 17°C. The IEF was performed by an Bio-Rad PROTEAN IEF Cell with the following parameters: (1) 100 V, 4 hours, slow; (2) 250 V, 2 hours, slow; (3) 500 V, 2 hours, slow; (4) 1000 V, 3 hours, rapid; (5) 10000 V, 5 h, liner; (6) 100000 V, 60000 vh, rapid; and (7) 500 V, 2 h, rapid. At the end of IEF, 0.375 M Tris-HCl equilibration buffer (1% DTT, 2% SDS, 20% glycerol, 6 M urea, pH 8.8) was applied to single strips. Iodoacetamide (2.5%) buffer was applied for another 15 mins to prepare the thiol-free groups. Strip proteins were dissolved in 10% SDS gels, and electrophoresis was conducted with Bio-Rad PROTEAN II xi Cell vertical electrophoresis bath system.

### 2.6. Silver Staining

Fixation liquid (methanol/water/acetic acid, 4/5/1 v/v) was applied to the gel for 1 h/overnight after electrophoresis, after which photosensitive solvents (3.14‰ sodium thiosulfate, 6.8% sodium acetate, 30% methanol) for 30 minutes. Every gel was rinsed by water for 3 times (each time for 5 minutes) and was stained under 2.50‰ silver nitrate for 20 minutes, and each gel was rinsed with water for 2 × 1 minutes and then improved by cultivating with the developer liquid (0.04‰ formaldehyde and 2.5% sodium carbonate) till development of the proteins. Then, silver staining was terminated by adding 14.6‰ EDTA for 10 min.

### 2.7. Gel Scanning and Image Analysis

A molecular Imager GS-800 Calibrated Densitometer (Bio-Rad, Hercules, CA, USA) was employed in this study for gel scanning and studied by PDQuest version 8.0 (Bio-Rad, Hercules, CA, USA). Image analysis includes site detection, site editing, background subtraction, and site matching. Comparisons were made between gel images of HCT-8/VCR cells with and without the treatment of CUR administration pair and pair. Proteins were sorted as being discriminatively expressed between the two cell lines when site intensity was showed a distinction ≥ twofold or sites which appeared or disappeared after dealing with 3 independent tests were chosen for analysis by HPLC-CHIP-MS/MS.

### 2.8. Mass Spectrometric Analysis of Proteins

Protein sites were isolated and every specimen was transferred to an EP tube (1.5 mL) and digested with trypsin (Sigma-Aldrich, St. Louis, MO, USA) in accordance with the manufacturer's statement. The MS of peptides were acquired by Bruker AutoflexIII MALDI-TOF/TOF MS (Bruker). Swiss-Prot 55.3 database (MASCOT 2.04 search engine, Matrix Science, UK) was employed for protein recognition with the following conditions: (i) enzyme, trypsin; (ii) type of search, MS/MS ion search; (iii) allowance of one missed cleavage; (iv) variable, oxidation of methionine; (v) fixed modification, carbamidomethylation; and (vi) fragment and peptide mass tolerance, 0.5 Da and 50 ppm. Among all the protein identifications, only those whose protein or ion score confidence intervals (CI) > 95% were accepted.

### 2.9. Western Blotting Analysis

Cells were dealt with using methods mentioned above. For separation of total protein draw, cells were rinsed by ice-cold PBS buffer solution and then lysed on ice in RIPA lysis buffer (1 mM EDTA, 1 mM PMSF, 50 mM Tris [pH 7.4]. 150 mM NaCl, 0.1% SDS, 1% Triton X-100, 1% sodium deoxycholate) for 10 minutes, after which centrifugation (12,000 rpm) was performed for the cell lysates under 4°C for 10 minutes. After gathering the supernatant, proteins in the drew samples were tested by BCA protein assay kit (Beyotime, Shanghai, China). Samples in this study were stored under -80°C for the next tests.

All cell lysate was heated in boiled water in 5 × loading buffer (0.5% bromchlorphenol blue, 8% DTT, 10% SDS, 50% glycerol, 125 mM Tris-HCl [pH 6.8]) for ten minutes. The same mass of proteins (30 *μ*g) was resolved by 12% SDS-PAGE and transferred to PVDF (Millipore, Billerica, MA, USA), which were thereafter obstructed by skim milk (5%) in TBST (0.1% Tween-20, 50 mM Tris-Cl [pH 7.6], 150 mM NaCl) under 37°C for two hours. Afterwards, coincubation of the membranes and primal polyclonal antibodies against glutathione S-transferase P1 (GSTP1), Peroxiredoxins 6 (PRDX6) (rabbit antihuman, Proteintech, Rosemont, IL, USA) and *β*-actin (rabbit anti-human, Santa Cruz) was carried out overnight under 4°C. After being washed using TBS including 0.05% Tween 20, the membranes were thereafter cultivated together with secondary antibody (rat antirabbit, Santa Cruz, Santa Cruz, CA, USA) for two hours. They were visual under chemiluminescence system on the basis of the manufacturer's statement.

### 2.10. Real-Time Reverse Transcription-Polymerase Chain Reaction (RT-PCR) Analysis

Cells would be treated as described above, all RNA of each cell line was separated using the RNAiso reagent (TaKaRa, Tokyo, Japan) on the basis of the manufacturer's agreement and soon afterwards converted into cDNA templates by reverse transcription using the SuperScript II Reverse Transcription kit (TaKaRa, Tokyo, Japan). The RT-PCR primers were conducted as follows: GSTP1 (forward primer 5′-GTAGTTTGCCCAAGGTCAAG-3′; reverse primer 5′-AGC CACCTGAGGGGTAAG-3′), PRDX6 (forward primer 5′-CACAAGCTTATGCCCGGAGGTCTGCTTC-3′; reverse primer 5′-CACGGTACCGTAGGCTGGGGTGTGTAG-3′). A PCR reaction was conA PCR reaction was conducted *β*-actin (forward primer 5′-TCCTGTGGGATCCACGAAACT-3′; reverse primer 5′-GAAGCATTTGCGGTGGACGAT-3′) for RNA normalization. Reactions were performed in accordance with the standard protocol. The reaction parameters were as follows: GSTP1, 5 minutes under 95°C, 1 minute under 94°C, 30 seconds under 59°C, and 1 minute under 72°C for 35 cycles, 72°C extension 5 minutes; PRDX6, *β*-actin, 3 minutes under 94°C, 30 seconds under 94°C, 30 seconds under 58°C, and 1 minute under 72°C for 35 cycles, 72°C extension 5 minutes. The 2% agarose gel electrophoresis was conducted for the resultant PCR products and visualized with EBr.

### 2.11. Statistical Analysis

The SPSS analytical software (v10.1, SPSS Inc., Chicago, IL, USA) was employed for all analysis in this study. All data would be presented in the formation of mean ± SEM, and the difference significance (treatment vs. control) was tested via Student's *t*-test. *P* levels < 0.05 suggested statistical significance.

## 3. Results

### 3.1. Cytotoxic Impacts of Curcumin on HCT-8/VCR Cells

**Figure 2:**
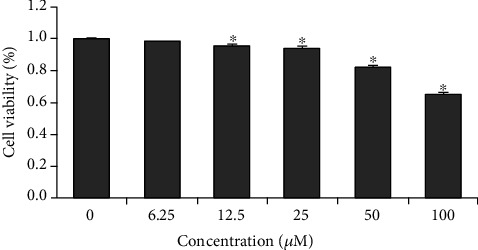
Impacts of CUR on HCT-8/VCR cell proliferation. Cells were handled with a variety of concentrations of curcumin (treatment groups) or DMSO (control groups) for 3 d. Viability was tested by MTT assay. Data from 4 independent tests were presented in the formation of Mean ± SEM (each treatment was conducted thrice). ^∗^*P* < 0.01 vs. the controls.

We discovered that 1-25 *μ*mol/L curcumin was not evidently cytotoxic to HCT-8/VCR cells (survival rate > 90%). But 50-100 *μ*mol/L curcumin caused prominent cytotoxicity in HCT-8/VCR cells (Figure 2). Because treatment of cells using curcumin of different dose levels (6.25, 12.5, 25 *μ*mol/L) had no prominent impact on cell viability, these concentrations (25 *μ*mol/L) were kept for further study.

### 3.2. Two-DE and MS/MS Analysis Results

**Figure 3:**
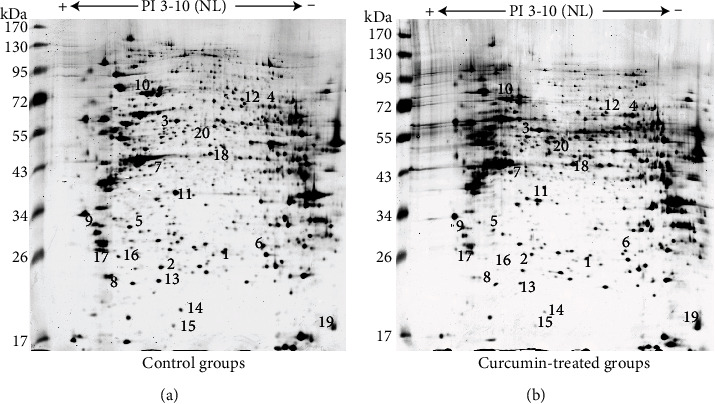
2-DE maps of control groups (a) and curcumin-treated groups (b). The *x*-axis is pI and the y-axis is molecular mass. Isolated proteins were visualized using silver staining. Twenty discriminatively showed proteins were indicated using circle and numbered by HPLC-CHIP-MS/MS. The corresponding identities are listed in Table 1.

**Figure 4:**
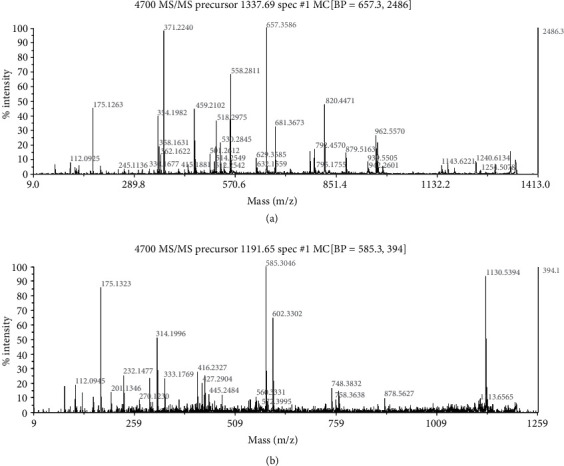
HPLC-CHIP-MS/MS assay of differential expression protein site 1 and 2. (a) HPLC-CHIP-MS/MS mass spectrum of site 1 discerned as the GSTP1 based on the matched peaks was indicated. (b) HPLC-CHIP-MS/MS mass spectrum of spot 2 identified as the PRDX6 according to the matched peaks was shown.

On the basis of the 2-DE, proteomic analysis was conducted to identify discriminatively protein expression before and after CUR treatment. To identify repeatability of results, two-DE was conducted thrice for all samples and analyzed using PDQuest version 8.0. The protein sites of the samples were mostly found in the gel in position referring to 26-95 kDa and the isoelectric points (PI) in the range of 3.5-8. Approximately, 1000 sites were tested on every gel using silver staining. Compared to the control groups, twenty-six proteins in total were obviously changed in groups treated with curcumin. A total of twenty protein sites were discerned by MALDI-TOF-MS (Figures 3 and 4). Three protein sites were upregulated in the curcumin-performed groups, and seventeen protein sites were downregulated. The information of the differential protein is listed in Table 1.

### 3.3. Expression Confirmation by Western Blotting

**Figure 5:**
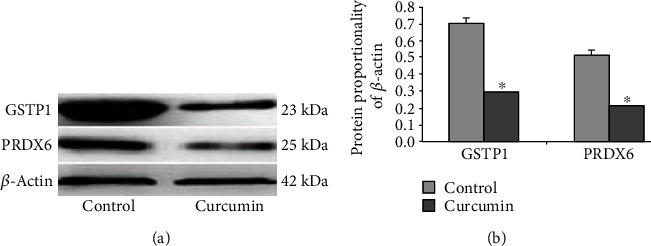
GSTP1 and PRDX6 proteins of different expression discerned by proteomic method and verified in Western blotting analysis. (a) Western blotting analysis of GSTP1 and PRDX6 protein levels in both cell lines, *β*-actin as a protein-loading control. Three independent test results were presented. (b) Normalized (relative to *β*-actin) GSTP1 and PRDX6 expressive levels. ^∗^*P* < 0.05 vs. the control.

To validate the identified proteins, we did Western blot analysis to verify two selected proteins, GSTP1 and PRDX6. As shown in Figure 5, GSTP1 and PRDX6 were decreased in the curcumin-treated HCT-8/VCR cells, consistent using the data shown in Figure 2 obtained using proteomic approach.

### 3.4. Expression Confirmation by RT-PCR

**Figure 6:**
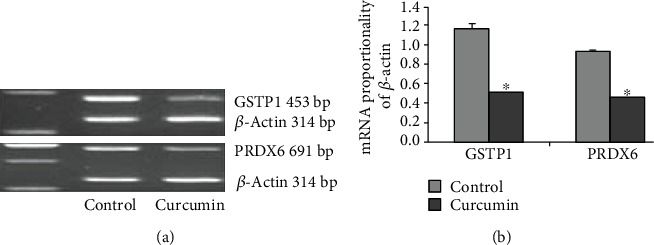
GSTP1 and PRDX6 proteins of different expression discerned by proteomic method and verified in RT-PCR. (a) RT-PCR analysis of mRNA expressive levels of GSTP1 and PRDX6. Data from 3 independent tests were presented in the formation of mean ± SEM. (b) Normalized (relative to *β*-actin) mRNA expressive levels of GSTP1 and PRDX6. ^∗^*P* < 0.05 vs. the control.

Expressions of mRNA for GSTP1 and PRDX6 were assessed by RT-PCR in this study. As indicated in Figure 6, GSTP1and PRDX6 were reduced in the curcumin-treated HCT-8/VCR cells, while similar decrease patterns were observed for mRNAs of GSTP1 and PRDX6 in the curcumin-treated HCT-8/VCR cells according to the two-DE and Western blotting analysis.

## 4. Discussion

Multidrug resistance (MDR) is generally acknowledged as cross-resistance to a wide spectrum of agents whose structures and functions are not necessarily related [18]. In the field of cancer treatment, MDR depicts the development of cross-resistance to various unexposed chemotherapeutic agents whose molecular structure and action mechanisms are different from the agent with resistance. Resistance to anticancer medicines is the main problem stopping the efficient treatment of cancer [19]. So, illustration of the mechanisms of MDR is of vital importance for the improvement of CRC prognosis. Curcumin mentioned above, a natural polyphenolic compound, can be applied to restrain main ABC drug transporters like P-gp, ABCG2, and MRP1 which are very important in the development of MDR [20–22]. But the famous multidrug resistance-related genes, MDR1, MRP1, LRP, and GST-*π* may not fully clarify the MDR [23].

This study applied high-throughput proteomics way to explore differences in the protein expression with the treatment of curcumin in HCT-8/VCR cells. As a reliable cell model, the well-resolved, renewable 2-DE profiles of MDR reversing of curcumin in HCT-8/VCR cells have been primarily established. Compared to those in HCT-8/VCR cell, twenty proteins were analyzed by HPLC-CHIP-MS/MS analysis. To identify the proteomic outcomes, 2 proteins GSTP1 and PRDX6 were chosen and verified by RT-PCR and Western blotting, the results of which were in line with the 2-DE gel image data, offering a rationale for the following functional explorations of selected proteins.

GSTs are important phase II detoxification enzymes with the biological function to catalyze the combination with glutathione of many electrophilic compounds [24, 25]. Mammalian GSTs have been divided into 5 different gene families: 4 cytosolic groups (Alpha, Mu, Pi, and Theta) and 1 microsomal form. GSTP1 is a member of the GST enzyme superfamily.

Overexpression of GSTP1 is widely observed in colorectal cancer, from aberrant crypt foci to advanced carcinomas [26]. Moreover, it has been discovered to be associated with medicine resistance in some carcinomas [27, 28] Andrew et al. [29] showed stable transfection of GSTP1 to HEp2 cells, which suggests involvement of GSTP1 enzymes and efflux mechanisms in the obtained doxorubicin-resistance phenotype. In addition, the cytotoxicity of doxorubicin was also improved after cell transfection with an antisense vector against GSTP1 [30]. Furthermore, by the administration of verapamil or increased doxorubicin concentration, the GSTP1 inhibitor curcumin improved in HEp2A cells as P-gp efflux obstacle had been reversed. What's more, curcumin had a chemo-sensitive impact under low doxorubicin [29]. Those prove that GSTP1 is an important factor in tumor cells for drug resistance. Therefore, downregulation of GSTP1 in curcumin-treated HCT-8/VCR cells should benefit the multidrug-resistance reversing of curcumin.

The Peroxiredoxins (PRDXs), thiol-containing peroxidases, a novel family of antioxidants which plays a critical role in breaking down hydrogen peroxide; thus, PRDXs can participate in cellular antioxidant defense [31]. PRDXs are also regulated cell proliferation differentiation apoptosis, chemotherapy [32–35] and radiotherapy resistance [36]. There are six isoforms of PRDXs (PRDX1–6) in mammal cells, which can be separated into two subgroups: the 2-Cys PRDXs and 1-Cys PRDXs [37–39]. PRDX6 is a key player in the removal of reactive oxygen species (ROS), but its characters clearly distinguish it from other family members. Because PRDX6 has a bifunctional protein with glutathione peroxidase and phospholipase A2 activities, which was named a “moonlighting” protein that is very important in physiology of antioxidant defense [39].

Young et al. [40] showed that gastric cancer cells were transfected with various concentrations of PRDX II antisense plasmid, pPRDXII/AS, and then administered with the same levels of cisplatin, PRDX II antisense enhanced cisplatin-induced cell death. In addition, Park et al. [41] also showed that conduct of 11A cells with a PRDX II antisense reduced induction of PRDX II, improving the radiation sensitivity. All these certify that PRDXs may play a certain role in tumor chemotherapy and radiotherapy resistance. It is conceivable that the decreased expression of PRDX6 in curcumin-treated HCT-8/VCR cells is an outcome of the PRDX6 impact of curcumin.

In summary, it is the first paper using the proteomic method to discern proteins MDR reversing of curcumin in HCT-8/VCR cells. Compared to the controls, a number of proteins were found to be significantly altered in curcumin-treated HCT-8/VCR cells. Our research suggested that GSTP1 and PRDX6 might take part in the MDR of human colon carcinoma. Among these proteins, some have not been studied to be relevant to carcinoma medicine resistance before, like PRDX6, and could be new multidrug-resistance relevant proteins. As a result, these discoveries could be a primary step to indicate the molecular mechanisms and make certain their roles and function requiring to be further researched in the future.

## 5. Conclusion

(1) In this study, HPLC-CHP-MS/MS was used for identification and bioinformatics analysis of twenty-six differentially expressed proteins, and a total of twenty proteins were identified. These differentially expressed proteins have an impact on biological characteristics of tumor cells such as division, apoptosis, signal transduction, glycolysis, multidrug resistance, and antioxidant

(2) The verification results of GSTP1 and PRDX6 protein expression levels were consistent with proteomics results

## Figures and Tables

**Table 1:** Information of differential expressed proteins in curcumin-treated HCT-8/VCR.

Spot No.	A spot	Protein name	Protein MW (Da)	Database point	Protein accession	Scores	Regulation
5105	1	PRDX6	25019.2	6	IPI00220301	441	Down
3102	2	GSTP1	23341	5.43	IPI00219757	415	Down
2506	3	FKBP4	51772.1	5.35	IPI00219005	71	Down
7701	4	XRCC5	82652.3	5.55	IPI00220834	108	Down
2101	5	ANXA5	35914.4	4.94	IPI00329801	74	Down
6103	6	HNRNPL	64092.4	8.46	IPI00027834	62	Down
2312	7	STOML2	38510.2	6.88	IPI00334190	86	Down
0112	8	TPT1	19582.6	4.84	IPI00550900	106	Down
0101	9	GSTP1	19468	5.67	IPI00793319	149	Down
3704	10	HSPA8	70854.2	5.37	IPI00003865	344	Down
3208	11	LDHB	36615.1	5.71	IPI0219217	309	Down
5711	12	STIP1	62599.4	6.4	IPI00013894	117	Down
3101	13	TPI1PI	26652.7	6.45	IPI00797270	616	Down
4002	14	NME1	30117.6	9.06	IPI00795292	88	Down
3008	15	SOD1	15925.9	5.7	IPI00218733	167	Down
1001	16	ARHGDIA	25815.4	6.93	IPI00796541	186	Down
0108	17	PSMA5	26394.2	4.74	IPI00291922	90	Down
4304	18	OAT	48504.2	6.57	IPI00022334	212	UP
9008	19	PPIB	23727.5	9.42	IPI00646304	66	UP
4406	20	HNRNPH1	49198.4	5.89	IPI00013881	96	UP

## Data Availability

On reasonable requests, datasets for analyses used in this study are available through contact with the corresponding author.
